# Transcriptional Expressions of ALDH1A1/B1 as Independent Indicators for the Survival of Thyroid Cancer Patients

**DOI:** 10.3389/fonc.2022.821958

**Published:** 2022-02-23

**Authors:** Ying Cui, Yao Liu, Lan Mu, Yang Li, Gang Wu

**Affiliations:** ^1^ Department of Otorhinolaryngology, The First Affiliated Hospital of Jinzhou Medical University, Jinzhou, China; ^2^ Department of Hepatobiliary Surgery, The First Affiliated Hospital of Jinzhou Medical University, Jinzhou, China

**Keywords:** ALDH1, thyroid cancer, prognostic biomarker, tumor-infiltrating lymphocytes, cancer progression

## Abstract

**Background:**

Aldehyde dehydrogenase (ALDH) 1 is an important enzyme involved in the regulation of several cellular mechanisms *via* aldehyde detoxification. High ALDH1 levels were correlated with tumorigenesis and stemness maintenance in cancer.

**Methods:**

We used UALCAN, Human Protein Atlas, Kaplan–Meier plotter, TISIDB, TIMER, and KOBAS databases to investigate the expression and role of ALDH1 in thyroid cancer progression. In addition, quantitative real-time polymerase chain reaction was performed to detect the expression of the target genes in thyroid cancer cell lines and cancer tissues.

**Results:**

Expression of ALDH1A1/B1 was significantly decreased based on individual cancer stages and tumor histology, and high levels of ALDH1A1/B1 were associated with poor overall survival in thyroid cancer patients. Moreover, ALDH1A1/B1 expression was negatively correlated with immune-stimulating genes, major histocompatibility complex, chemokines, and receptors.

**Conclusions:**

These results suggest that ALDH1A1/B1 might serve as potential prognostic biomarkers for thyroid cancer diagnosis.

## Introduction

Thyroid cancer is the most prevalent endocrine malignancy worldwide, with a global incidence of over 500,000 per year (1). The number of endocrine cases has been growing rapidly for decades (2), resulting in approximately 90% of the endocrine malignancies and 70% of deaths (3). Thyroid cancer was reported to be prevalent in women (4), but more advanced-stage cases were found in men (5). Recently, different research groups have explored prognostic markers and therapeutic targets for thyroid cancer, and some progress has been made (1, 4, 6). However, current diagnosis of thyroid cancer is still far from sufficient owing to the lack of appropriate biomarkers.

The aldehyde dehydrogenase 1 (ALDH1) family consists of conserved enzymes that oxidize aldehydes to carboxylic acids (7). The ALDH1 family includes six members: ALDH1A1 to ALDH1A3, ALDH1B1, ALDH1L1, and ALDH1l2 (8), which are localized in cytosol and mitochondria and can be found in almost all important organs (8). Recent studies demonstrated that Nuclear factor E2-related factor 2 (Nrf2) activation is involved in cancer stem cell-like properties’ exposure to high ALDH1A1 concentrations (9) that result in shorter overall survival (OS) and progression-free survival in ovarian cancer patients (10). In addition, targeting ALDH1 may facilitate the elimination of cancer stem cells and inhibit breast cancer progression (11, 12). Cumulative evidence has revealed that aberrant expression of ALDH1 is being evaluated as a potential novel prognostic marker in breast cancer (13), head and neck squamous cell carcinoma (7), ovarian cancer (9), and lung cancer (14). However, the role of the ALDH1 family in thyroid cancer remains poorly understood.

In this study, we systematically investigated the role of the ALDH1 family in thyroid cancer patients by searching public databases. We demonstrated that the levels of ALDH1A1/B1 had been significantly decreased in thyroid cancer patients. Aberrantly elevated ALDH1A1/B1 levels in cancer tissues were negatively correlated with OS in thyroid cancer patients. Besides, the perturbation of ALDH1A1/A3/B1 expression was significantly correlated with pathological stage. Finally, we elucidated the important but overlooked role of ALDH1A1/B1 in tumor-infiltrating lymphocytes (TILs), and we revealed a non-negligible immune regulatory role of ALDH1A1/B1 by drawing gene expression heat maps. Overall, our data provide motivation to better understand the tumor promotion and immune inhibition role of ALDH1A1/B1 in thyroid cancer patients, suggesting that ALDH1A1/B1 might serve as potential prognostic biomarkers for thyroid cancer treatment.

## Methods

### Correlation Between Target Genes and Pathological Stages

The Gene Expression Profiling Interactive Analysis (GEPIA) dataset is a web-based tool exploited by Tang et al. (15) to analyze the RNA sequencing (RNA-seq) expression data in cancer tissues and normal samples. Here, we used GEPIA to investigate the target genes’ expression level in thyroid cancer tissues and normal control and analyzed the relationship between target genes and pathological stage in thyroid cancer patients. And search term was limited to thyroid cancer. In addition, target proteins expressed in thyroid cancer tissues were confirmed by immunohistochemistry data acquired from The Human Protein Atlas (https://www.proteinatlas.org/).

### UALCAN

UALCAN (http://ualcan.path.uab.edu) is a free web database based on The Cancer Genome Atlas (TCGA) level 3 RNA-seq and 31 different types of cancer (16). In the current study, we investigated the expression level of target genes in thyroid cancer tissues and corresponding adjacent tissues based on UALCAN platform, and the search term was limited to thyroid cancer. Finally, we analyzed data based on individual cancer stages, patient’s gender, patient’s age, tumor histology, and nodal metastasis. P-value was determined based on Student’s t-test, and P < 0.05 was considered as statistically significant.

### Survival Analysis

Initiation and progression of many cancers could be characterized by aberrant change of gene expression (17). Here, we used Kaplan–Meier plotter (http://kmplot.com/analysis/) to access the prognostic value of ALDH1 level in thyroid cancer patients. Kaplan–Meier plotter can investigate the gene expression level in pan-caner under TCGA consortium support (18). We investigated expression levels of target genes in thyroid cancer tissues and corresponding adjacent tissues, and the search term was limited to thyroid cancer. Finally, we analyzed data based on patient’s gender to plot the OS, first progression (FP), and post progression survival (PPS) of patients with thyroid cancer, with the hazard ratio (HR) (95% confidence intervals) and log rank P-value.

### Analysis of Target Genes in Tumor-Infiltrating Immune Cells

Tumor IMmune Estimation Resource (TIMER, https://cistrome.shinyapps.io/timer/) contains 10,897 tumors from 32 cancer types (19), which could be used to systematically analyze the association between the target genes and tumor-infiltrating immune cells. Here, we investigated the somatic copy number alterations of target genes with the Somatic copy number alterations (SCNA) module. And correlation between target genes and infiltrating immune cells also was analyzed by limiting the search term to thyroid cancer.

In addition, the correlation between target genes with lymphocytes and immunomodulators was investigated by using the TISIDB database (http://cis.hku.hk/TISIDB/index.php).

### Construction of Protein–Protein Interaction

inBio Discover (https://inbio-discover.com/) could visualize protein–protein interaction networks under genome scale in humans (20). inBio Discover contains over 500,000 interactions from 8 source databases. Here, we used inBio Discover to construct target gene networks by limiting the search terms to ALH1A1, ALDH1A3, and ALDH1B1.

### Cell Culture and Sample Collection

Thyroid epithelial cell line nthy-ori 3-1 and thyroid cancer cell lines (CHO-K1 and TPC-1) were purchased from the Shanghai Zhong Qiao Xin Zhou Biotechnology Co., Ltd. Cell lines were mycoplasma negative, and nthy-ori 3-1 was cultured in RPMI 1640 medium, and thyroid cancer cell lines were cultured in Dulbecco’s modified Eagle’s medium (DMEM). All cell lines were supplemented with 10% fetal bovine serum (Gibco, USA).

Five thyroid cancer tissues and their corresponding adjacent tissues were collected from the First Hospital of Jilin University and stored at -80°C conditions. All participants were given written informed consent, and the current study has been approved by the ethics committee of the First Hospital of Jilin University.

### Quantitative Real-Time PCR

We used TransZol Up (Transgen, CN) to extract total RNA from the cell lines and cancer tissues according to manufacturer’s instructions. cDNA was synthesized by using the Hifair^®^ II 1st Strand cDNA Synthesis Kit (Yeasen, CN) according to manufacturer’s instructions. Then, quantitative real-time PCR (qRT-PCR) was performed using a Hieff^®^ qPCR SYBR Green Master Mix (Yeasen, CN) according to the manufacturer’s instructions. Here, β-actin was used as endogenous control. The primer sequence sets were listed as follows: β-actin: F: 5′TTCAACACCCCAGCCATG3′, R: 5′CCTCGTAGATGGGCACAGT3′; ALDH1A1: F: 5′ CCGTGGCGTACTATGGATGC3′, R: 5′GCAGCAGACGATCTCTTTCGAT3′; ALDH1B1: F: 5′AGAGTCTTACGCCTTGGACTT3′, R: 5′ GTCTTGCCATGCCACTTGTC3′.

### Statistical Analysis

Significance was determined with the either HR and P-values for plotting survival curves. The independent-samples Student’s t-test analysis was performed on GraphPad Prism 7.0, and P < 0.05 was considered statistically significant.

## Results

### Aberrant Expression of the ALDH1 Family in Thyroid Cancer Patients

**Figure 1:**
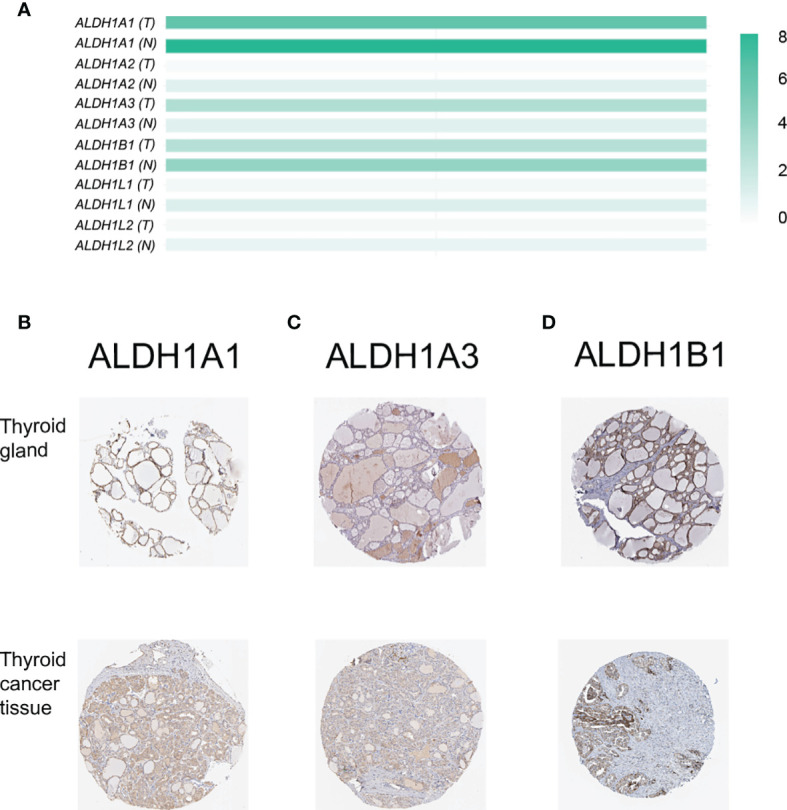
Confirmation of the expression of ALDH1 family members in thyroid cancer tissues. **(A)** ALDH1A1/A3/B1 were the top 3 genes expressed in thyroid cancer tissues (T, tumor tissues, n = 503; N, normal tissues, n = 59). **(B–D)** ALDH1A1/A3/B1 expression was confirmed by immunohistochemistry data from The Human Protein Atlas. Data in panel **(A)** were collected from the Gene Expression Profiling Interactive Analysis (GEPIA) database. Data in panels **(B–D)** were collected from The Human Protein Atlas database.

The members of the ALDH1 family have been proposed as potential therapeutic targets of ovarian cancer (21) and have shown the prognostic value in lung cancer (14) and breast carcinomas (22). However, the role of the ALDH1 family in thyroid cancer is still ill-defined. Here, we investigated the expression of the ALDH1 family in thyroid cancer, and we found that all ALDH1 family members could be detected in thyroid cancer tissues at the transcriptional level (**Figure 1A**). Interestingly, the expressions of ALDH1A1 and ALDH1B1 were higher in the normal thyroid tissues and decreased in the thyroid cancer tissues (**Figure 1A**). In contrast, the level of ALDH1A3 was slightly increased in cancer tissues (**Figure 1A**). The low expression levels of other genes in the thyroid tissues were negligible. Furthermore, immunohistochemical results verified that ALDH1A1, ALDH1A3, and ALDH1B1 were highly expressed in thyroid cancer at the protein level (**Figures 1B**–**D**), suggesting the role of ALDH1A1/A3/B1 in thyroid cancer development. Therefore, we determined the expressions of ALDH1A1/A3/B1 in thyroid cancer tissues.

### ALDH1A1 and ALDH1B1 Expression Decreased in Thyroid Cancer Patients

**Figure 2:**
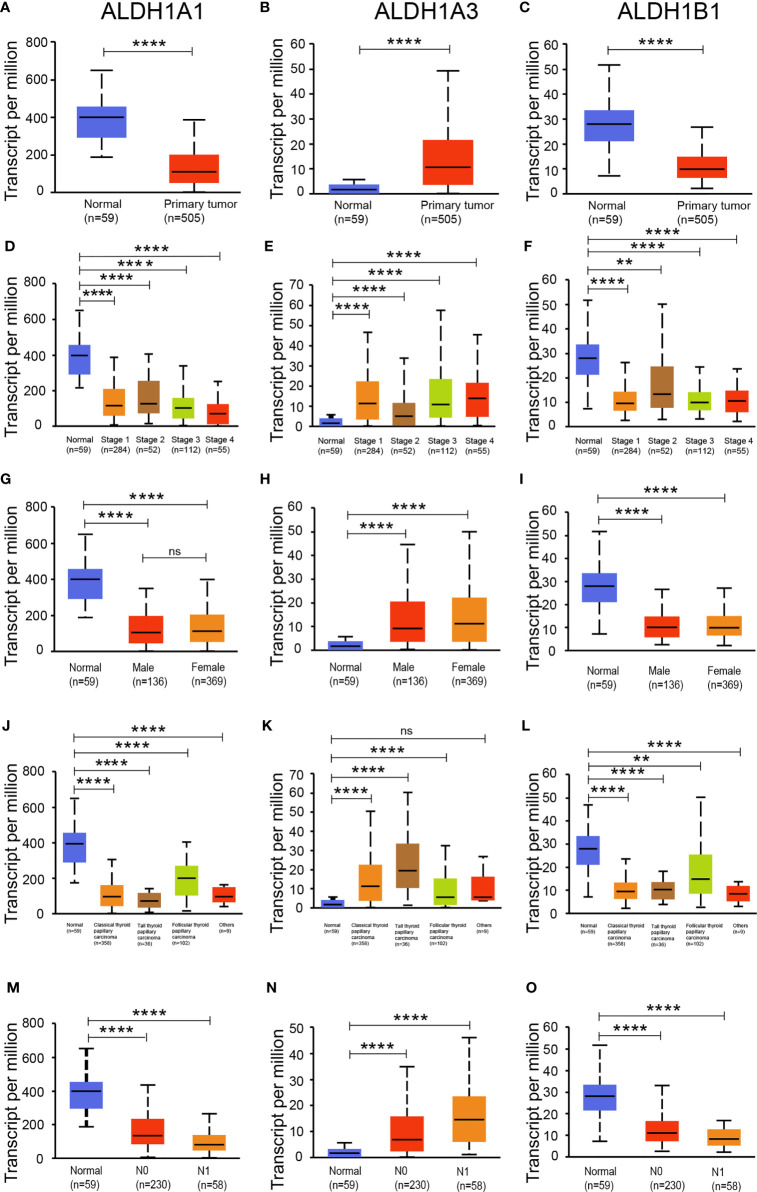
Expression of ALDH1A1/B1 was significantly decreased in thyroid cancer tissues. Differential expression of ALDH1A1 **(A)**, ALDH1A3 **(B)**, and ALDH1B1 **(C)** in thyroid cancer tissues and normal tissues based on individual cancer stage **(D–F)**, patient’s gender **(G–I)**, histological subtype **(J–L)**, and nodal metastasis status **(M–O)** (N0, no regional lymph node metastasis; N1, metastases in 1–3 axillary lymph nodes; N2, metastases in 4–9 axillary lymph nodes; N3, metastases in 10 or more axillary lymph nodes). **P < 0.01; ****P < 0.0001. Data were collected from the UALCAN database. ns, No significance.

As the expressions of ALDH1A1/A3/B1 were relatively high in thyroid cancer tissues, we assessed the level of ALDH1A1/A3/B1 in thyroid tumors and normal tissues. Our results showed that the transcriptional levels of ALDH1A1 and ALDH1B1 significantly decreased in thyroid cancer tissues, whereas that of ALDH1A3 increased (**Figures 2A**–**C**). Next, we further assessed the changes in ALDH1A1/A3/B1 based on different classifications. As shown in **Figure 2**, mRNA expressions of ALDH1A1 and ALDH1B1 significantly decreased in thyroid cancer tissues compared to that in normal groups based on individual cancer stages (**Figures 2D**–**F**), gender (**Figures 2G**–**I**), tumor histology (**Figures 2J**–**L**), and nodal metastasis (**Figures 2M**–**O**). Meanwhile, the levels of ALDH1A3 significantly increased (**Figures 2B, E, H, K, N**). These results indicate that ALDH1A1/A3/B1 may be correlated with and may play a significant role in thyroid cancer progression.

### Expressions of ALDH1A1/A3/B1 Were Correlated With the Pathological Stage in Thyroid Cancer

**Figure 3:**
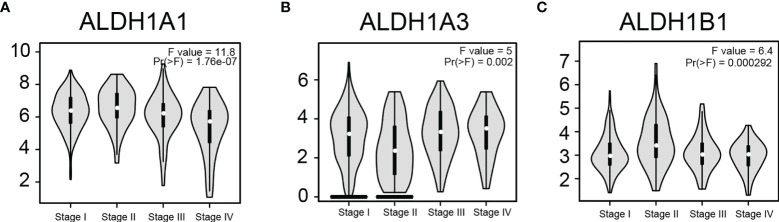
Correlation between differentially expressed ALDH1A1/A3/B1 and the pathological stage of thyroid cancer patients. Violin plots showed the distribution of ALDH1A1/A3/B1 [**(A–C)**, respectively] mRNA expression correlated with tumor stage in thyroid cancer patients (Stage I, n = 284; stage II, n = 52; stage III, n = 112; stage IV, n = 55). Data were collected from the Gene Expression Profiling Interactive Analysis (GEPIA) database.

To determine if ALDH1A1/A3/B1 is associated with tumor progression and clinical outcome of thyroid cancer, we assessed the correlation between the expression of ALDH1A1/A3/B1 and the pathological stage of the patients. Results showed that there was a significant correlation between the expression of ALDH1A1, ALDH1A3, and ALDH1B1 with the pathological stage (**Figures 3A**–**C**). These results indicate that the aberrant expression of ALDH1A1/A3/B1 may correlate with tumorigenesis and development.

### Prognostic Value of mRNA Expression of ALDH1A1/A3/B1 in Thyroid Cancer Patients

**Figure 4:**
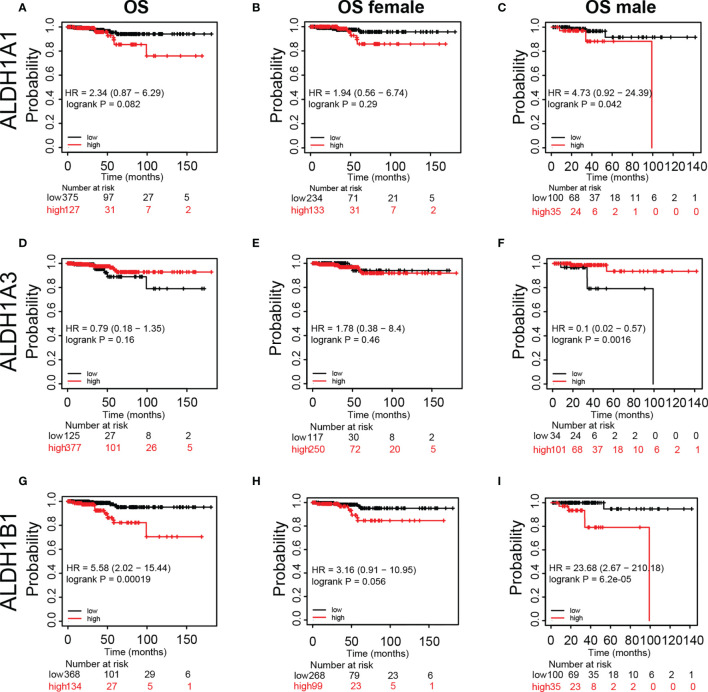
Kaplan–Meier plot revealing the correlation between ALDH1A1/A3/B1 level and overall survival (OS) in thyroid cancer patients. Patients were divided into two groups based on ALDH1A1/A3/B1 level [**(A, D, G)**, respectively], and Kaplan–Meier plot of ALDH1A1/A3/B1 in female patients **(B, E, H)** and male patients **(C, F, I)** with thyroid cancer (total number of patients with thyroid cancer, n = 502; total number of female patients with thyroid cancer, n = 367; total number of male patients with thyroid cancer, n = 135). Data were collected from the Kaplan–Meier plotter database.

Furthermore, we assessed the prognostic significance of ALDH1A1/A3/B1 in patients using Kaplan–Meier plotter analysis. As shown in **Figure 4**, patients were divided into low- and high-risk groups based on the expression levels of ALDH1A1/A3/B1. Our results showed that changes in the expression level of ALDH1A1/A3 had no influence over the OS of female patients with thyroid cancer (**Figures 4A, B, D, E**); however, increased mRNA level of ALDH1A1 impaired OS in male patients with thyroid cancer (**Figure 4C**). Moreover, high mRNA expression of ALDH1A3 only enhanced OS in male patients with thyroid cancer (**Figure 4F**). And high levels of ALDH1B1 also impaired OS in patients with thyroid cancer (**Figures 4G**–**I**). Taken together, we identified that ALDH1A1/A3/B1 in thyroid cancer patients were significantly correlated with OS in male patients, supporting the possible prognostic value of these enzymes particularly for male patients with thyroid cancer.

### A Significant Correlation Between ALDH1A1/A3/B1 Expression and Tumor-Infiltrating Immune Cells (Tumor-Infiltrating Lymphocytes) in the Tumor Microenvironment

**Figure 5:**
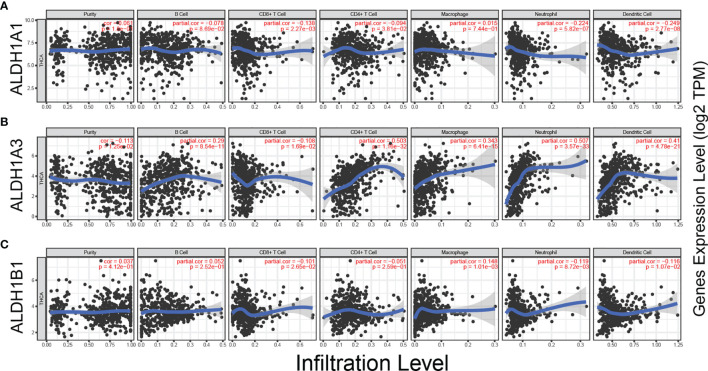
Correlation between ALDH1A1/A3/B1 expression and immune infiltration in thyroid cancer. **(A–C)** Correlation of ALDH1A1/A3/B1 expression with infiltration levels of CD8^+^ T cell, CD4^+^ T cell, Treg cell, B cell, neutrophil, macrophage, dendritic cell, natural killer cell, and monocyte in thyroid cancer (total number of patients with thyroid cancer, n = 223). Data were collected from the Tumor IMmune Estimation Resource.

Tumor-infiltrating lymphocytes (TILs) are shaped by interactions between different types of killer cell, regulatory cell, stroma cell, and tumor cell in the tumor microenvironment (23). Recent studies have shown that tumor progression or suppression is closely associated with TIL response in the tumor microenvironment (24). Thus, we investigated the relationship between ALDH1A1/A3/B1 expression and TILs using TIMER. ALDH1A1/B1 showed weak correlation with the six key types of immune cell in the tumor microenvironment (**Figures 5A, C**). Meanwhile, a significant correlation between ALDH1A3 expression and neutrophils, CD4^+^ T cells, dendritic cells, macrophages, and B cells (**Figure 5B**) was observed. These results suggest that ALDH1A1/A3/B1 may affect multiple cells in the tumor microenvironment that may participate in the tumor immune response.

### ALDH1A1/A3/B1 Broadly Interacted With Multiple Proteins

**Figure 6:**
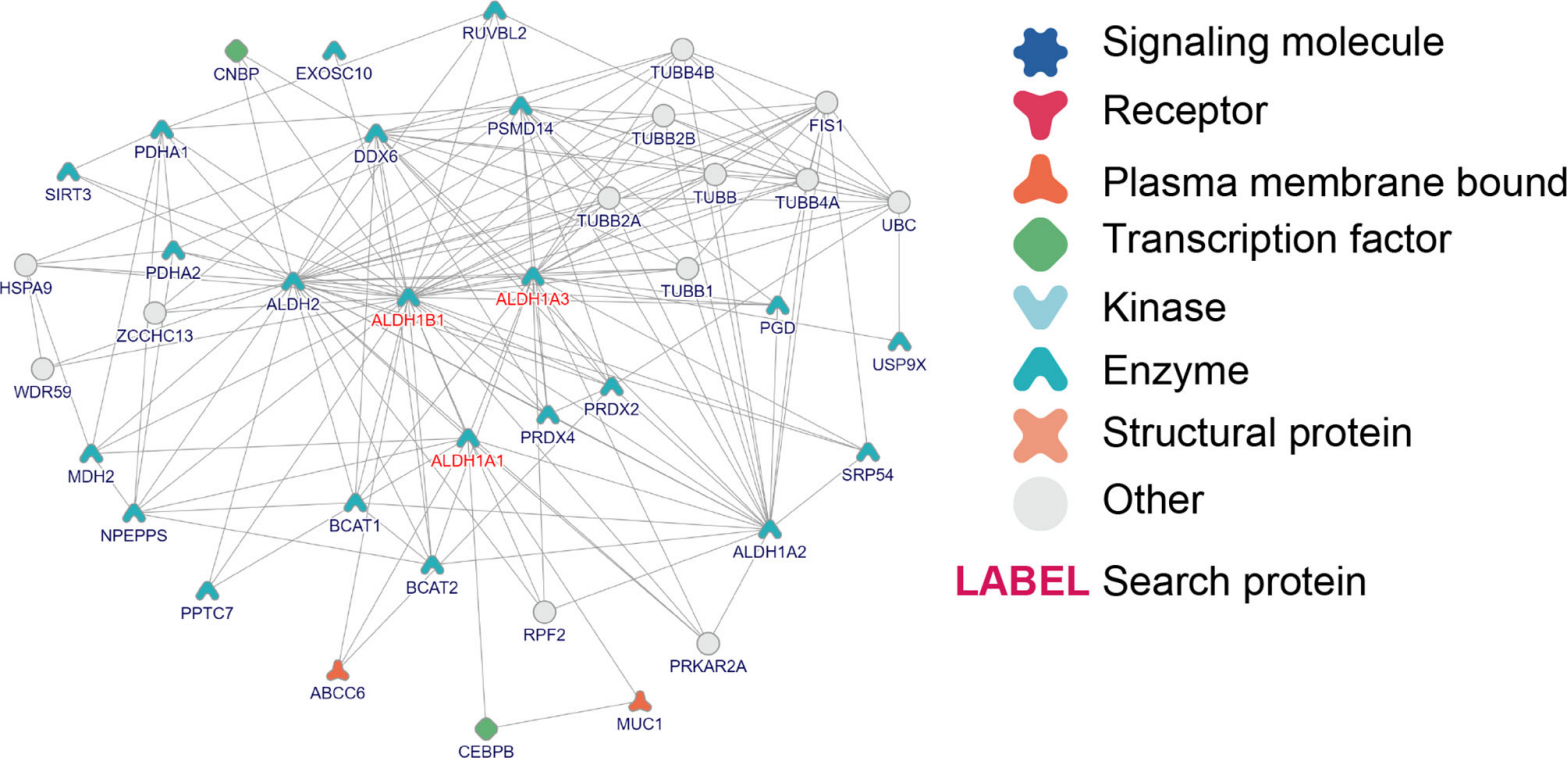
Interaction and Gene Ontology (GO)/pathway analysis of ALDH1A1/A3/B1. Protein–protein interaction network of ALDH1A1/A3/B1, constructed by the online tool inBio Discover.

Furthermore, to study the role of ALDH1A1/A3/B1 in thyroid cancer, we constructed protein–protein interaction networks by using the tool of inBio Discover. The results showed that ALDH1A1/A3/B1 could interact with various proteins (**Figure 6**), most of which were identified as enzymes. Besides, we showed that ALDH1A1 and ALDH1B1 could interact with transcription factors [CCAAT/enhancer-binding protein β (CEBPB) and CCHC-type zinc finger nucleic acid-binding protein (CNBP), respectively] and membrane-bound proteins [ATP-binding cassette subfamily C member 6 (ABCC6) and mucin 1 (MUC1)], suggesting that the aberrant changes of ALDH1A1/B1 may alter multiple protein expressions and intracellular signal transduction. Indeed, our Gene Ontology (GO) enrichment analysis revealed a close relationship between ALDH1A1/A3/B1 and ethanol oxidation (GO:0006069), as well as retinol metabolic process (GO:0042572), which are known to be important in detoxification (8), tumorigenesis (25), and thyroid tumor progression (**Supplementary Material**) (26). Specifically, we found that ALDH1A1/A3/B1 were enriched in thyroid hormone binding (GO:0070324). Similarly, Kyoto Encyclopedia of Genes and Genomes (KEGG) pathway analysis also showed that ALDH1A1/A3/B1 were associated with metabolic pathways (**Supplementary Material**). Collectively, these results indicate that ALDH1A1/A3/B1 may influence thyroid cancer *via* metabolism and interaction with various enzymes and transcription factors.

### Aberrant Levels of ALDH1A1/A3/B1 Are Correlated With Immune Molecular Regulation in Thyroid Cancer

**Figure 7:**
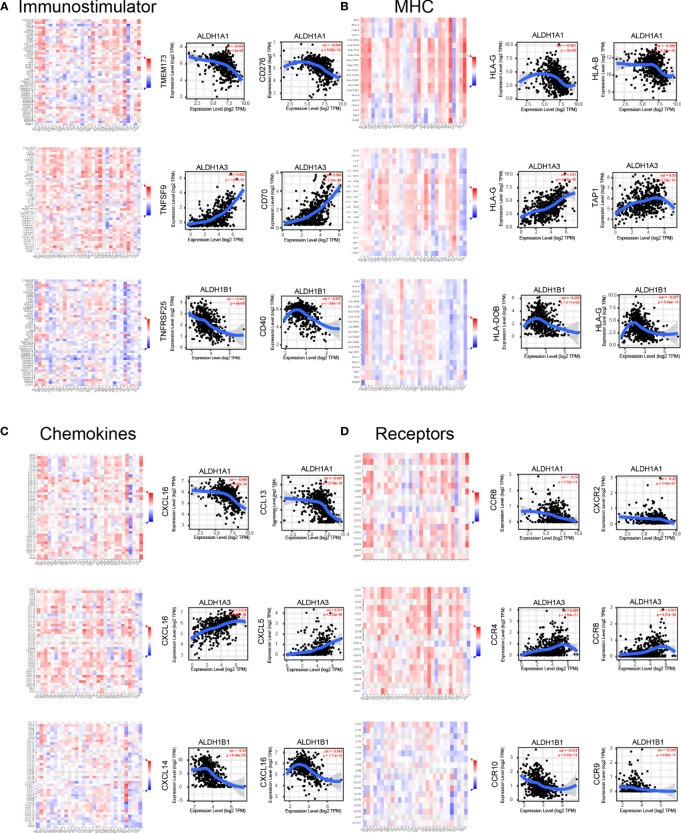
Correlation of ALDH1A1/A3/B1 with lymphocytes and immunomodulators. Relations between the abundance of immunostimulators **(A)**, MHC **(B)**, chemokines **(C)**, and receptors **(D)** and ALDH1A1/A3/B1 expression and the top 2 genes displaying the greatest Spearman’s correlation with ALDH1A1/A3/B1 expression. Red and blue cells indicate positive and negative correlations, respectively. The color intensity is directly proportional to the strength of the correlations. Data were collected from the TISIDB and TIMER database (total number of patients with thyroid cancer, n = 223). TILs, tumor-infiltrating lymphocytes, MHC major histocompatibility complex; TIMER, Tumor IMmune Estimation Resource.

Immune modulation is a critical factor that should be considered in cancer therapy, as numerous genes play a role in response to cancer immunotherapy (27). Using the TISIDB database, we demonstrated that ALDH1A1/A3/B1 had significant negative correlations with immune-stimulating genes, such as CD276, TMEM173, TNFSF9, CD70, TNFRSF25, and CD40 in thyroid cancer (**Figure 7A**). Moreover, high levels of ALDH1A1/B1 were also associated with restrained expression of major histocompatibility complex (MHC) genes (HLA-G, HLA-B, HLA-DOB, and TAP1) (**Figure 7B**). Notably, previous studies have demonstrated that higher levels of immune stimulators and MHC promote better therapeutic effect in cancer patients (28, 29). In addition, the expressions of ALDH1A1/B1 were found to be negatively correlated with chemokines (CXCL5, CXCL16, and CCL13; and CXCL14, respectively) (**Figure 7C**) and receptors (CXCR2, CCR4, and CCR8, and CCR9, CCR10, respectively) (**Figure 7D**). Therefore, ALDH1A1/B1 may be involved in regulating the above immune molecules.

### ALDH1A1/B1 Expressions Significantly Decreased in Thyroid Cancer Cell Line and Cancer Tissues

**Figure 8:**
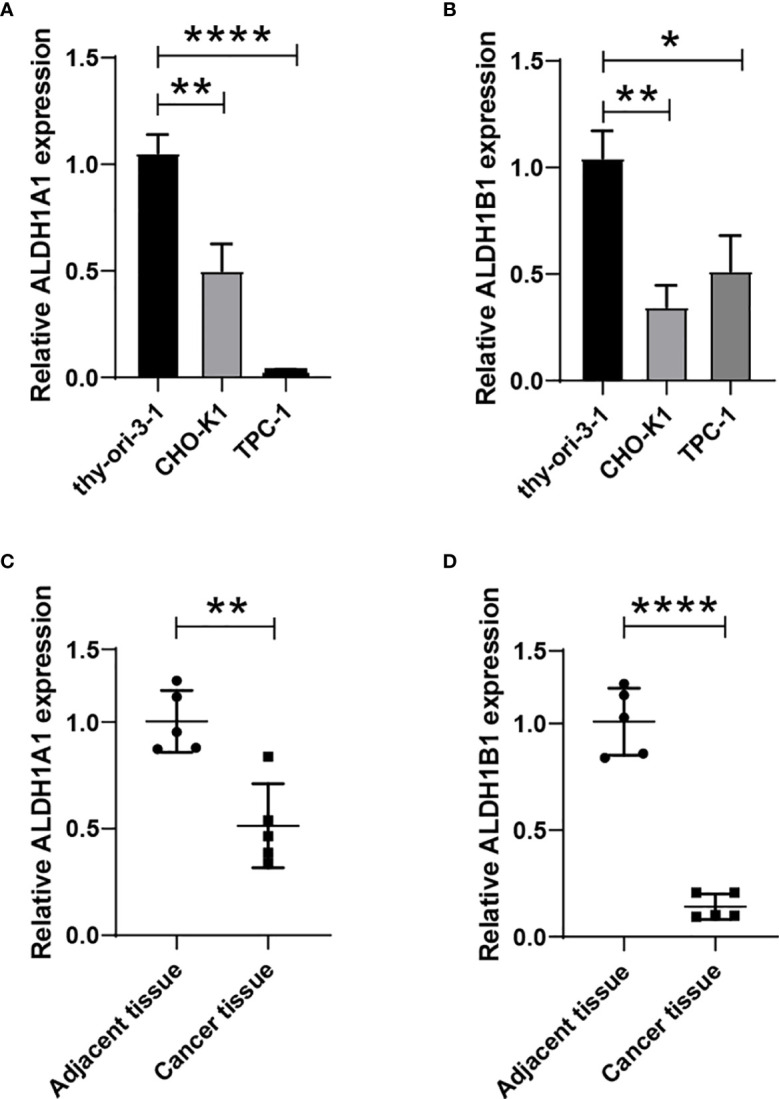
Reduced expression of ALDH1A1/B1 in thyroid cancer cell lines and thyroid cancer tissues. Thyroid epithelial cell line nthy-ori 3-1 and thyroid cancer cell lines (CHO-K1 and TPC-1) were collected, and the levels of ALDH1A1 **(A)** and ALDH1B1 **(B)** were detected by using qRT-PCR. Five thyroid cancer tissues and corresponding adjacent tissues were collected from the First Hospital of Jilin University to verify the expression of ALDH1A1 **(C)** and ALDH1B1 **(D)** in patients. *P < 0.05; **P < 0.01; ****P < 0.0001.

We have observed a decreased expression of ALDH1A1/B1 in thyroid cancer tissues (**Figures 1A**–**D**), which was correlated with poor survival of patients (**Figures 4C, G, I**). Thus, we aimed to verify the levels of ALDH1A1/B1 in thyroid cancer cell lines and thyroid cancer tissues *in vitro*. We found that ALDH1A1/B1 expression was significantly reduced in CHO-K1 and TPC-1 cells (**Figures 8A, B**). In addition, we also detected a decreased expression of ALDH1A1/B1 in thyroid cancer tissues (**Figures 8C, D**). These results confirmed the effectiveness and reliability of the results in bioinformatic analysis.

## Discussion

ALDH1 is a critical gene mediating the enzymatic detoxification of aldehydes (8), and it is also required in cell proliferation (30) and stem cell differentiation (31) in cancers. Although all six ALDH1 members have been reported to be intracellularly involved in various biological processes, ALDH1A1/A3/B1 were the highly expressed genes in thyroid cancer tissues in this study. We observed that the regulatory functions of ALDH1A1 and ALDH1B1 in thyroid cancer were involved in poor outcome in patients and inhibition of TILs.

ALDH1 has been reported to play a role in promoting triple-negative breast cancer progression and enhanced drug resistance in a Notch receptor 4 (NOTCH4)-dependent manner (32, 33). Ciccone et al. (31) observed that overexpression of ALDH1A1 was significantly associated with Vascular endothelial growth factor (VEGF)-mediated angiogenesis by retinoic acid/ Hypoxia-inducible factor (HIF)-1α signal. Similarly, a study on esophageal cancer demonstrated that ALDH1 was positively correlated with higher clinical stage and poor prognosis (34). Here, we showed that ALDH1A1/B1 expressions were significantly decreased in thyroid cancer patients, but ALDH1A3 was elevated. However, the level of ALDH1A3 had no significant changes. These results indicated the different roles of ALDH1 members in thyroid cancer progression. ALDH1A1 could regulate retinoid signaling *via* the CEBPB to promote stemness maintenance in cancer stem cells (35). In addition, ALDH1A1 also profoundly affected acetaldehyde metabolism and drug resistance in chemotherapy (8), suggesting that ALDH1A1 could broadly regulate biological processes in cancer. Indeed, our results showed that elevated ALDH1A1 was negatively correlated with OS of thyroid cancer patients in male patients but was beneficial to Relapse-free survival (RFS) in female patients; it indicated that differently expressed gonadal hormones may also be involved in ALDH1A1 regulation in thyroid cancer development.

Although the tumorigenic property of ALDH1A3 in cancer has been observed (8, 36), it is suggested that upregulated epithelial-to-mesenchymal transition and cancer stem cell-like properties related genes, including ALDH13, may be involved in the lymphatic spread of papillary thyroid microcarcinoma (37). However, another study found that administration of ALDH inhibitor had no impact on proliferation or spherogenicity in several thyroid cancer cell lines (38). Here, we showed that aberrant expression of ALDH1A3 was not associated with OS in women. Meanwhile, we noted that there was a significant correlation between the expression of ALDH1A3 with the pathological stage in patients. These data suggest that increased ALDH1A3 may only affect alternative subtypes of thyroid cancer. However, the role of ALDH1A3 in thyroid cancer development still needs further study.

Upregulation of ALDH1B1 by Kras is associated with pancreatic lineage homeostasis and initiation/development of pancreatic ductal adenocarcinoma, which are characteristics of enhanced production of reactive oxygen species by altering mitochondrial metabolism (39). ALDH1B1 is an enzyme that has been proposed to play an important role in acetaldehyde detoxification. Increased ALDH1B1 level was involved in the regulation of the Wnt-, Notch-, and Phosphatidylinositol-3-kinase (PI3K)/Akt signaling pathways in colon tumorigenesis (40). In this study, high ALDH1B1 expression in thyroid cancer was associated with poor OS in male patients, suggesting a correlation between ALDH1B1 and androgen. Notably, a study in prostate cell lines showed that cells with elevated ALDH1B1 expression frequently expressed androgen receptor (41). In addition, a study in prostate cancer cell line LNCaP reported that androgen dihydrotestosterone was involved in aldehyde dehydrogenase regulation (42). Also, a high level of aldehyde dehydrogenase promoted metastatic growths in stem-like prostate cancer cells (43). Therefore, androgen receptor expression may be involved in ALDH1B1-induced thyroid cancer progression.

We have shown the negative correlation between ALDH1A1/B1 and several immune-stimulating genes. In addition, we observed an opposite expression of the ALDH1A1/B1 and MHC genes. During thyroid cancer progression, the immune response relies on MHCs to recognize and present antigens to cytotoxic T lymphocytes. Low levels of immune-stimulating genes and MHCs would inhibit immune cell activation and induce a weaker response or promote immunological unresponsiveness (44). Recently, several chemokines were reported to be highly expressed in thyroid cancer tissues, which might be involved in cancer progression and invasion by activating Akt and Erk signaling pathway (45). Our results revealed the importance of chemokines and receptors in thyroid cancer, which were in agreement with the study that emphasized the crucial role of chemokine receptors during papillary thyroid carcinoma development (45). As soluble mediators secreted by cells in tumor microenvironments, different chemokines recruit various types of immune cells by binding to its corresponding receptors (46). Moreover, targeting chemokine and chemokine receptor has been implicated in non-small cell lung cancer (47), gastric cancer (48), and breast cancer therapy (49). Of note, our results showed a negative correlation between ALDH1A1/B1 and several chemokines/receptors in thyroid cancer tissues. Thus, some chemokines/receptors that showed decreased expression in thyroid cancer tissues may be avoided as potential therapeutic targets.

In conclusion, we have revealed the aberrant expressions of ALDH1A1/B1 in thyroid cancer tissues that were significantly correlated with the OS of patients and with the pathological stage. In addition, we found that elevated ALDH1A1/B1 expression was negatively associated with chemokine/receptor expression and involved in several TILs. Thus, ALDH1A1/B1 are potential biomarkers for thyroid cancer and might be valuable therapeutic targets for diagnosis and therapy. However, future studies are required to validate our findings and promote the clinical utility of ALDH1A1/B1 in thyroid cancer treatment.

## Data Availability Statement

The datasets used and/or analyzed during the present study are available from the corresponding author on reasonable request.

## Ethics Statement

All participants were given written informed consent, and the current study has been approved by the ethics committee of the First Hospital of Jilin University. The patients/participants provided their written informed consent to participate in this study.

## Author Contributions

YC performed the experiments. YC and YLiu analyzed the data and wrote the article. LM collected samples. YC, YLiu, YLi, and GW revised the article. GW conceived the idea and supervised the project. All authors contributed to the article and approved the submitted version.

## Funding

This work was supported by Scientific Research Funds of the Education Department of Liaoning Province (JYTQN2020018).

## Conflict of Interest

The authors declare that the research was conducted in the absence of any commercial or financial relationships that could be construed as a potential conflict of interest.

## Publisher’s Note

All claims expressed in this article are solely those of the authors and do not necessarily represent those of their affiliated organizations, or those of the publisher, the editors and the reviewers. Any product that may be evaluated in this article, or claim that may be made by its manufacturer, is not guaranteed or endorsed by the publisher.
