# Supplementary material for: Transcriptional Expressions of ALDH1A1/B1 as Independent Indicators for the Survival of Thyroid Cancer Patients
Source: Front Oncol. 2022 Feb 23;12:821958. doi: 10.3389/fonc.2022.821958 (PMC8905520; doi:10.3389/fonc.2022.821958)
Supplement: Supplementary file 1 [file Table_1.docx]

Table 1 GO analysis of ALDH1A1/A3/B1 associated genes with thyroid cancer.

| Term | Description | Count | *p*-value | adj p-values |
| --- | --- | --- | --- | --- |
| GO:0006069 | ethanol oxidation | 12 | 0.000000354 | 0.00000437 |
| GO:0042572 | retinol metabolic process | 47 | 0.00000458 | 0.0000339 |
| GO:0001758 | retinal dehydrogenase activity | 8 | 0.000000175 | 0.00000324 |
| GO:0051287 | NAD binding | 39 | 0.00000319 | 0.0000295 |
| GO:0004030 | fructose catabolic process to hydroxyacetone phosphate and glyceraldehyde-3-phosphate | 5 | 0.000458575352271 | 0.00205653616259 |
| GO:0004030 | aldehyde dehydrogenase [NAD(P)+] activity | 5 | 0.000458575352271 | 0.00205653616259 |
| GO:0043878 | glyceraldehyde-3-phosphate dehydrogenase (NAD+) (non-phosphorylating) activity | 6 | 0.000534990944991 | 0.00205653616259 |
| GO:0055114 | oxidation-reduction process | 525 | 0.000535087975537 | 0.00205653616259 |
| GO:0060013 | righting reflex | 7 | 0.000611402642931 | 0.00205653616259 |
| GO:0070324 | thyroid hormone binding | 7 | 0.000611402642931 | 0.00205653616259 |

Table 2 KEGG pathway analysis of ALDH1A1/A3/B1 related pathways with thyroid cancer.

| Term | Description | Count | *p*-value | adj p-values |
| --- | --- | --- | --- | --- |
| hsa00340 | Histidine metabolism | 23 | 0.00000117 | 0.00000958 |
| hsa00010 | Glycolysis / Gluconeogenesis | 68 | 0.0000094 | 0.0000482 |
| hsa00410 | beta-Alanine metabolism | 33 | 0.00000232 | 0.0000158 |
| hsa05204 | Chemical carcinogenesis | 82 | 0.00633 | 0.00683 |
| hsa00980 | Metabolism of xenobiotics by cytochrome P450 | 76 | 0.00587 | 6.51e-3 |
| hsa00982 | Drug metabolism - cytochrome P450 | 72 | 0.00557 | 0.00634 |
| hsa00830 | Retinol metabolism | 67 | 0.00519 | 0.00608 |
| hsa00561 | Glycerolipid metabolism | 61 | 0.00473 | 0.00571 |
| hsa00310 | Lysine degradation | 59 | 0.00458 | 0.00569 |
| hsa00330 | Arginine and proline metabolism | 50 | 0.00389 | 0.00499 |
